# TRP channels in epileptogenesis: calcium dysregulation mechanisms and pharmacological targeting strategies

**DOI:** 10.3389/fnmol.2025.1687359

**Published:** 2025-09-25

**Authors:** Guolong Deng, Dayuan Liu, Yunxiang Zhong, Muyao Wang, Baoshou Su, Hongli Jiang, Yihao Zhai, Hao Peng, Caicai Zhang, Jigao Feng

**Affiliations:** ^1^Department of Neurosurgery, The Second Affiliated Hospital of Hainan Medical University, Haikou, China; ^2^Key Laboratory of Brain Science Research Transformation in Tropical Environment of Hainan Province & Key Laboratory of Tropical Translational Medicine of Ministry of Education, College of Basic Medical Sciences, Hainan Medical University, Haikou, China; ^3^Department of Neurosurgery, Hainan Affiliated Hospital of Hainan Medical University, Haikou, China

**Keywords:** TRPV, TRPM, TRPC, epilepsy, therapeutic targets

## Abstract

Epilepsy, a prevalent neurological disorder affecting millions globally, manifests as recurrent synchronous neuronal discharges that disrupt normal cerebral function. Emerging evidence characterizes this condition as a network-level hyperexcitability disorder driven by aberrant neuroelectrical synchronization. At the molecular level, intracellular calcium (Ca^2+^) overload is increasingly recognized as a key contributor to seizure initiation and propagation. The regulation of neuronal Ca^2+^ homeostasis involves multiple Ca^2+^ − permeable cation channels, with transient receptor potential (TRP) channels emerging as critical mediators of pathological ion flux. These non-selective transmembrane conduits facilitate Ca^2+^ permeation and contribute to epileptogenic ionic dysregulation through subtype-specific mechanisms. Current research efforts focus on elucidating TRP channel pathophysiology across epilepsy subtypes while identifying potent pharmacological modulators. This systematic investigation of TRP channel biology and targeted therapeutic development promises to revolutionize antiepileptic drug discovery by addressing current treatment limitations in seizure prevention and disease modification. The present review synthesizes recent advances in TRP channel research and evaluates emerging strategies for therapeutic targeting in epilepsy management.

## 1 Introduction

Epilepsy constitutes a prevalent neurological disorder characterized by enduring predisposition to epileptic seizures. These seizures manifest as transient episodes of hyper-synchronous pathological neuronal hyperactivity, resulting in acute disruption of normative cerebral network functionality. Such electrophysiological aberrations generate objectively discernible neurological signs and/or subjective symptom complexes (Kanner and Bicchi, 2022; Asadi-Pooya et al., 2023). Epilepsy affects approximately 65 million individuals worldwide, corresponding to a prevalence rate of 6.38 per 1,000 people (Devinsky et al., 2018). Current research identifies the most common causes of neurological disorders as genetic factors, neurotransmitter imbalances, metabolic abnormalities, ion channel dysfunction, synaptic alterations, inflammatory and immune dysregulation, and structural and functional changes (Thijs et al., 2019). Epilepsy is defined by a chronic predisposition to unprovoked seizures, manifesting as a clinical spectrum ranging from focal aware seizures to generalized tonic–clonic convulsions (García-Rodríguez et al., 2022). Epileptic seizures are classified as focal or generalized, depending on whether abnormal neuronal activity originates within a localized region of one hemisphere or involves widespread bilateral cortical networks (Devinsky et al., 2018). Patients with epilepsy exhibit a 2–10% reduction in life expectancy compared to the general population (Moshe et al., 2015). Patients with epilepsy face a fourfold elevated risk of depression and anxiety disorders compared to the general population (Salpekar and Mula, 2019). Frequent or prolonged seizures may induce severe cognitive impairment, memory deficits, and neuropsychiatric comorbidities (Ding et al., 2021). These sequelae can irreversibly disrupt adolescent neurodevelopment, substantially diminishing quality of life while imposing significant socioeconomic burdens on patients, families, and healthcare systems (Ding et al., 2021).

Significant advances have been achieved in epilepsy research and therapeutic development by the early 21st century, including the introduction of novel anti-seizure medications (ASMs) (Carvill et al., 2020). Nevertheless, over one-third of patients develop drug-resistant epilepsy, remaining refractory to available pharmacological interventions (Rugg-Gunn et al., 2020; Duncan and Taylor, 2023). Contemporary ASMs primarily function through modulation of voltage-gated ion channels, potentiation of GABAergic inhibition, attenuation of glutamatergic excitation, and presynaptic regulation of neurotransmitter release. However, most existing agents terminate ongoing seizures without modifying the underlying epileptogenic processes, thereby failing to prevent epileptogenesis or alter the natural history of epilepsy (Galanopoulou et al., 2021; Löscher, 2020). Consequently, elucidating epileptogenic mechanisms to identify novel therapeutic targets represents a critical imperative for developing disease-modifying strategies that may overcome these limitations, ultimately improving patient outcomes and alleviating the societal burden. Mounting evidence implicates dysregulated calcium signaling in the pathophysiological cascade of epileptogenesis (Lv et al., 2011). Intracellular calcium homeostasis is regulated by multiple calcium-permeable cation channels, with transient receptor potential (TRP) channels representing particularly promising therapeutic targets. Consequently, elucidating the pathophysiological roles of TRP channels in epilepsy may accelerate the development of novel disease-modifying therapies.

## 2 TRP channel biology and pathology

TRP channels, named after the Drosophila ‘transient receptor potential’ mutant first described in 1969 (Cosens and Manning, 1969), are polymodal cation channels ubiquitously expressed across diverse tissues and cell types. Functioning as sensors for physicochemical stimuli (Wu et al., 2010; Nilius and Szallasi, 2014), these channels primarily localize to plasma membranes as non-selective cation channels, whose permeability to Ca^2+^ can regulate Ca^2+^ release from intracellular organelles, thereby modulating critical physiological processes (Koivisto et al., 2022). Across mammalian species, the TRP channel superfamily consists of 28 genes, though only 27 functional orthologs exist in the human genome (Wu et al., 2010; Wang R. et al., 2020). Based on variations in amino acid sequences and topological structures, the TRP ion channel superfamily is classified into seven major subfamilies: canonical transient receptor potential canonical (TRPC) (TRPC1–7), vanilloid transient receptor potential vanilloid (TRPV) (TRPV1–6), melastatin transient receptor potential melastatin (TRPM) (TRPM1–8), ankyrin transient receptor potential ankyrin (TRPA) (TRPA1), polycystin transient receptor potential polycystin (TRPP) (TRPP2–3), mucolipin transient receptor potential mucolipin (TRPML) (TRPML1–3), and NO-mechano-potential transient receptor potential NO-Mechano-Potential (TRPN) (Wu et al., 2010; Wang R. et al., 2020). Some channels are activated by heat (TRPM2/4/5, TRPV1-4), while others are activated by cold (TRPA1, TRPC5, and TRPM8) (Baez et al., 2014; Brauchi and Orio, 2011). TRPN is only found in invertebrates and fish, and is not expressed in mammals (Wang R. et al., 2020). TRP channels can form functional channels as homotetrameric or heterotetrameric complexes within the same or different subfamilies (Pizzo et al., 2001). The prototypical TRP channel architecture comprises six transmembrane *α*-helical segments (S1-S6), flanked by an intracellular N-terminal domain and an extended C-terminal cytoplasmic region (Liedtke and Heller, 2006). Transmembrane domains can be divided into two components: S1-S4 form a pseudo voltage-sensing domain, and S5-S6 form a pore domain composed of helices and pore helices. These pores form ion conduction pores, which act as selective filters in TRP channels. The pseudo voltage-sensing-like domain (S1-S4) senses stimuli, transmits information to the gate, and causes conformational changes (Liedtke and Heller, 2006). While high-resolution structures of full-length TRP channels remain limited, current models derived from cryo-EM analyses suggest a sophisticated architecture. A notable feature is the interaction between adjacent subunits, where the voltage-sensing-like domain (VSLD) of one subunit engages in a ‘domain-swapped’ conformation with the pore domain (PD) of its neighbor. This intricate arrangement is proposed to be crucial for allosteric coupling, whereby structural changes in the VSLD in response to stimuli are efficiently transmitted to the central pore gate, thereby modulating channel activity. Transmembrane domains exhibit high sequence homology within a specific subfamily, while the intracellular N-terminal domain and C-terminal regions show lower homology between subfamilies. These regions are variable in length and sequence and possess distinct domains and motifs, contributing to the functional and structural diversity of the protein across different subfamilies (Cabezas-Bratesco et al., 2022; Li et al., 2011; Pan et al., 2011).

TRP channels are responsible for various sensory responses, including heat, cold, pain, pressure, vision, and taste, and can be activated by many stimuli (Zhang et al., 2023). Abnormal expression and function of TRP channels are associated with various diseases, such as metabolic diseases, chronic pain, neurological diseases, cardiovascular diseases, respiratory diseases, and renal diseases (Zhao et al., 2021; Li et al., 2017). TRP channels are widely distributed in neurons and the brain and are thought to be associated with neurological disorders (Kuppusamy et al., 2021; Sisignano et al., 2014). They are associated with neurological disorders such as epilepsy, stroke, dementia, anxiety, neurodegenerative diseases, and depression (Sisignano et al., 2014).

Research has found that TRPC channels are associated with various neurological diseases. Specific inhibition of TRPC1 and TRPC5 can suppress the activation of extracellular regulatory protein kinase/cyclic adenosine monophosphate response element-binding protein and provide neuroprotective effects (Yao et al., 2009). TRPC1 overexpression also exerts a protective effect against Parkinson’s disease by mediating calcium influx, inhibiting the release of cytochrome C from mitochondria, and reducing 1-methyl-4-phenyl-1,2,6-tetrahydropyridine-induced neurotoxicity and apoptosis. In Alzheimer’s disease, TRPC1, TRPC3, and TRPC4 have been implicated in neuroprotective pathways (Kim et al., 2006; Li et al., 1999), whereas TRPC3 may contribute to tau protein dysregulation and TRPC5 has been associated with neurodegenerative processes (Vaidya et al., 2023; Yamamoto et al., 2007). Behavioral data from a human study of TRPC6 have found that its deficiency in mice leads to reduced exploratory behavior, which may be associated with autism (Griesi-Oliveira et al., 2015). Research has also revealed structures of TRPA1 bound to the agonist iodoacetamide and the antagonist A-967079, identifying a potentially conserved Ca^2+^ regulatory site (Brauchi and Rothberg, 2020). The toxic effects of amyloid-*β* on astrocytes triggered by TRPA1 channel activation are a key factor in the progression of Alzheimer’s disease (Paumier et al., 2022). TRPA1 is closely associated with anxiety and depressive behavior (De Moura et al., 2014). Under hypoxic conditions, endothelial TRPA1 channels cause vasodilation (Taylor-Clark, 2016), thereby reducing ischemic damage. This series of phenomena is thought to be related to ischemic stroke (Pires and Earley, 2018). The study also found that TRPM dysfunction is involved in the process of various neurological diseases. TRPM2 and TRPM7 are located downstream of multiple signaling pathways in the oxidative stress response induced by cerebral ischemia–reperfusion injury, which is considered a key factor leading to neuronal death (Hara et al., 2002). In experimental rodent models of cerebral ischemia, TRPM4 expression was upregulated in the endothelium of the penumbra. Blocking TRPM4 promoted vascular formation on the matrix gel and improved vascular integrity after *in vitro* oxygen/glucose deprivation (Loh et al., 2014). Additionally, research has confirmed that TRPVs play a role in the development of various neurological disorders. Modulating the opening channels on the surface of microglia can enhance autophagy and phagocytosis in these cells, thereby improving the condition of patients with Parkinson’s disease (Yuan et al., 2022). The activation of TRPV1 channels on astrocytes initiates endogenous neuroprotective mechanisms *in vivo*, thereby preventing the activity of dopamine neurons from changing (Nam et al., 2015). TRPV4 mediates endoplasmic reticulum stress and inflammatory pathways, leading to the loss of dopaminergic neurons in the substantia nigra and motor deficits in Parkinson’s disease mice (Liu et al., 2022). In an Alzheimer’s disease mouse model, the metabolic-enhancing effects of TRPV1 agonists reduced amyloid lesions and reversed memory impairment (Lu et al., 2021). TRPV4 inhibitors enhanced the expression of the neurogenic marker doublecortin and increased the levels of the mature neuron marker NeuN in mice with cognitive impairment, suggesting that TRPV4 is closely associated with dementia characterized by impaired learning and memory (Deng et al., 2022). Activation of TRPV2 can induce the release of neurotrophic factors and regulate blood–brain barrier function, which may be related to ischemic stroke (Luo et al., 2019). Since TRP channels are more widely distributed in the nervous system, their exact functions require further investigation. Studying the relationship between different TRPs and neurological disorders is of great significance for the diagnosis and treatment of these diseases. Research has also shown that TRP channels allow cation influx, increasing intracellular free Ca^2+^ concentration, leading to membrane depolarization and enhanced excitability (Nilius, 2013), This may also be one of the reasons why multiple TRP channels are involved in epilepsy.

## 3 The role of TRPV ion channels in epilepsy

Within the TRPV family, TRPV1 is one of the most characteristic and relatively well-studied ion channels (Yang et al., 2023). Each TRPV1 subunit has six transmembrane regions (TM), and four TRPV1 subunits form a channel that is permeable to monovalent and divalent cations, with a single-channel conductance of 50–100 picosiemens (Tsagareli and Nozadze, 2020). Research has found that TRPV1 is involved in the pathophysiological processes of many diseases, such as epilepsy, cerebral ischemia, schizophrenia, etc. (Díaz-Franulic et al., 2016; Gladkikh et al., 2021). Similar to other TRP channels, TRPV1 is a non-selective cation channel with high permeability to Ca^2+^ (Caterina et al., 1997). TRPV1 was first discovered in sensory neurons, and subsequent studies have confirmed its important role in inflammatory pain (Caterina et al., 1997). Subsequent studies have also confirmed that TRPV1 is expressed in the cell bodies and dendrites of neurons in the hippocampus, cortex, olfactory bulb, midbrain, and cerebellum, with significantly higher expression in the hippocampus and cortex (Mezey et al., 2000; Kim et al., 2020; Senn et al., 2020). This indicates a close relationship between TRPV1 and epilepsy (Table 1).

**Table 1:** Interaction between TRPV and Epilepsy.

TRPV subfamily	Interaction	References
TRPV1	TRPV1 activation promotes seizure genesis via axonal hyperexcitability; pharmacological blockade suppresses ongoing seizures	Gonzalez-Reyes et al. (2013)
	Capsaicin-induced TRPV1 activation promotes dentate gyrus apoptosis and seizures	Manna and Umathe (2012)
TRPV4	TRPV4 activation (Ca^2+^-dependent) promotes seizure-associated neuroinflammation and neuronal injury via ER stress; its inhibition reduces glial activation and damage	Wang et al. (2019)
	Epileptic hyperthermia at foci amplifies TRPV4/TRPV1 activation, accelerating pathogenesis	Shibasaki et al. (2020)

Research has found that activation of TRPV1 channels leads to increased brain excitability in mice, while inhibiting their activity can effectively suppress epileptic seizures (Gonzalez-Reyes et al., 2013). TRPV1 channel blocker capsazepine can effectively reduce the susceptibility to epilepsy in a rat model of hereditary epilepsy and also reduce the severity of epileptic seizures (Cho et al., 2018). Another TRPV1 antagonist, N-arachidonoyl-5-hydroxytryptamine (AA-5-HT), can effectively inhibit pentylenetetrazine-induced seizures in mice and shorten seizure duration (Vilela et al., 2014). In epileptic rats, activation of TRPV1 by capsaicin leads to apoptosis of hippocampal dentate gyrus cells, highlighting the important role this channel plays in regulating seizures (Manna and Umathe, 2012). In contrast, TRPV1 antagonists showed a protective effect on hippocampal neurons in the same rat epilepsy model (Nazıroğlu and Övey, 2015). Similarly, intraperitoneal administration of the TRPV1 antagonist capsazepine or TRPV1 gene knockout delayed the latency of tonic–clonic seizures in mice and reduced the mortality rate in the pentylenetetrazole-induced epilepsy model (Jia et al., 2015). Consistent with this, in Wistar rats, the selective TRPV1 agonist N-oleoyldopamine increased the total incidence of pentylenetetrazole-induced seizures. Conversely, following administration of the TRPV1 antagonist AMG-9810, the severity and duration of seizures in the rat amygdala kindling model were significantly reduced (Shirazi et al., 2014). Additionally, the study found that capsaicin, when co-administered with the exogenous cannabinoid receptor agonist WIN 55,212–2, reduces its anticonvulsant effects. In contrast, co-administration of capsazepine enhances the inhibitory effect of WIN 55,212–2 on the activation response of the dentate gyrus, supporting a potential association between TRPV1 and cannabinoid signaling in brain disorders associated with excessive excitability (Carletti et al., 2016). Furthermore, a descriptive human study revealed elevated TPRV1 mRNA and protein expression in the cortex and hippocampus of patients with medial temporal lobe epilepsy relative to controls. TRPV1 was predominantly expressed in neuronal cell bodies and dendrites, with no significant expression detected in astrocytes or microglia (Sun et al., 2013).

However, contrary to the aforementioned promotion of seizure onset by TRPV1, studies have also found that congenital TRPV1 deficiency increases the susceptibility of newborn mice to pentylenetetrazole-induced seizures after repetitive hyperthermia challenges, suggesting that TRPV1 plays an important role in the pathogenesis of febrile seizures in newborns (Kong et al., 2014). The use of TRPV1 antagonists capsazepine or AMG-9810 alone for systemic treatment can reduce pentylenetetrazole-induced seizures in mice in a dose-dependent manner, but simultaneously reduces the anticonvulsant effect of acetaminophen, suggesting that acetaminophen may exert its anticonvulsant effect through TRPV1 (Suemaru et al., 2018). In a lithium chloride/piracetam rat epilepsy model, relatively low doses of capsaicin treatment exacerbated hippocampal neuronal death and reversed the neuroprotective effects of dexmedetomidine in immature rats, suggesting that TRPV1 activation may lead to neuronal death induced by epilepsy in young animals (Tan et al., 2020). In summary, whether TRPV1 activation leads to beneficial or harmful effects appears to depend on various factors, including the epilepsy model, animal age, and particularly its cell type-specific distribution. These pharmacological studies suggest that selective inhibition of TRPV1 channels may represent a new therapeutic strategy for epilepsy.

**Figure 1:**
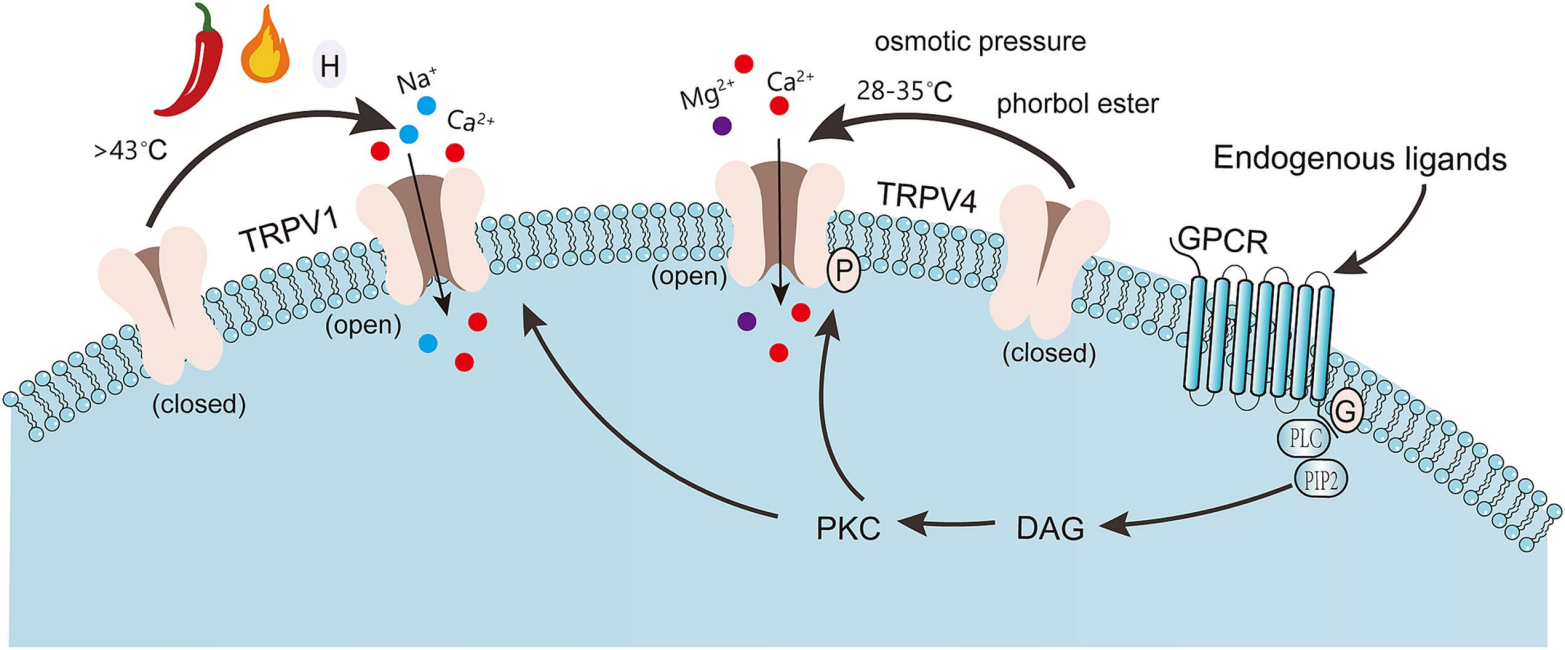
Capsaicin, pH, and high temperatures can activate TRPV1, primarily through the activation of the cAMP-dependent PKA pathway. The PKC-dependent cascade is also involved. TRPV4 is activated by moderate heat (28–35 °C), osmotic pressure, and phorbol esters (plant-derived compounds). Some endogenous ligands, such as epinephrine, adenosine, and dopamine, activate G protein-coupled receptors to stimulate the PLC pathway, inducing the hydrolysis of PIP2 into DAG. DAG activates PKC, which in turn phosphorylates TRPV1/4 channels. TRPV1: Transient Receptor Potential Vanilloid 1. TRPV4: Transient Receptor Potential Vanilloid 4. DAG: Diacylglycerol. PKC: Protein Kinase C.

TRPV4 channels are non-selective cation channels that allow the passage of ions and small molecules and are primarily expressed in mammals (Karasawa et al., 2008). In the brain, TRPV4 is mainly expressed in neurons and glial cells (Cao et al., 2009; Kanju and Liedtke, 2016). The activation and inactivation of TRPV4 depend on Ca^2+^, and the activation of TRPV4 channels allows Ca^2+^ to enter the cell, causing endoplasmic reticulum stress and regulating cell excitability (Wang et al., 2019). In animal models of epilepsy, TRPV4 expression is increased in the hippocampus (Men et al., 2019), Additionally, elevated intracranial temperatures in epilepsy were found to result in more pronounced activation of TRPV4 and TRPV1 (Shibasaki et al., 2020). The study also found that activation of TRPV4 increases neuroinflammation and neuronal damage following seizures (Wang et al., 2019). In addition, the application of TRPV4 antagonists can significantly reduce neuronal damage and the activation of astrocytes and microglia in a temporal lobe epilepsy animal model (Wang et al., 2019). Research has found that young zebrafish larvae exhibit upregulation of TRPV4 expression following high-temperature-induced seizures, and that RN-1734 inhibits seizures by suppressing TRPV4 (Hunt et al., 2012). Lithium chloride/pirocarperol-induced epilepsy in mice showed high expression of TRPV4 in the hippocampus, and administration of the TRPV4 antagonist HC-067047 suppressed epileptic seizures in mice (Men et al., 2019). In this study, TRPV4 agonists increased the mRNA and protein levels of gap junction protein 43, which plays an important role in the onset and development of epileptic seizures. The upregulation of gap junction protein 43 expression by TRPV4 is associated with the occurrence of epilepsy (Men et al., 2019). The study also found that intracerebroventricular injection of the TRPV4 agonist GSK1016790A in mice led to upregulation of interleukin-1β, glial proliferation, and ultimately neuronal death in the hippocampus. In contrast, intracerebral injection of the TRPV4 antagonist HC-067047 significantly improved seizures in pilocarpine-induced epileptic rats and mitigated post-seizure glial proliferation and cytokine activation (Wang et al., 2019). GSK1016790A activates TRPV4, which enhances the expression of voltage-gated potassium channel Kv4.2 and potassium channel-interacting protein in mouse hippocampal tissue induced by piracetam, while the TRPV4 inhibitor HC-067047 attenuates the expression of this protein (Xu et al., 2022). The elevation of TRPV4-mediated Kv4.2 and potassium channel interaction proteins are believed to lead to an increase in fast inactivating potassium currents in hippocampal pyramidal neurons, which may contribute to excessive excitation in the early stages of epilepsy (Xu et al., 2022). Following 4-phenylaminopyridine-induced seizures in mice, TRPV4 and glial fibrillary acid protein were both upregulated in the hippocampus, and the colocalization of these two proteins suggests that the upregulation of TRPV4 may depend on the activation of astrocytes (Zeng et al., 2022). HC-067047 inhibits TRPV4 and reduces the susceptibility of mice to 4-phenylaminopyridine-induced seizures and several key pro-inflammatory mediators, while treatment with GSK1016790A increases the mortality rate in epileptic animal models (Zeng et al., 2022). These studies suggest that targeting TRPV4 activation in astrocytes may provide new therapeutic strategies for acute seizures and epilepsy progression (Figure 1).

## 4 The role of TRPC ion channels in epilepsy

The TRPC channel is considered the primary TRP cation channel, responsible for capacitive Ca^2+^ influx and involved in the pathophysiological functions of various neurological disorders (Lee et al., 2021). TRPC channels are involved in various neuropathophysiological processes, including neuronal excitability, neurogenesis, axonal growth, excitotoxicity, neuronal apoptosis, and necrotic cell death, as well as neurodegeneration (Wang H. et al., 2020; He et al., 2017; Jeon et al., 2020). The activation of TRPC channels facilitates the influx of Ca^2+^ and Na^+^, leading to the further release of Ca^2+^ from the endoplasmic reticulum via ryanodine receptors in the form of Ca^2+^ transients. This process is referred to as Ca^2+^-induced Ca^2+^ release (Del Prete et al., 2014). The release of Ca^2+^ ultimately leads to membrane depolarization and increased cytoplasmic Ca^2+^ concentration, playing a crucial regulatory role in the cell (Wang H. et al., 2020) (Table 2).

**Table 2:** Interaction between TRPC and epilepsy.

TRPC subfamily	Interaction	References
TRPC1/4/5	TRPC1/4 drive seizure bursts; TRPC5 modulates LTP in epileptogenesis, with KO conferring excitotoxic protection	Phelan et al. (2013)
TRPC3/6	Status epilepticus upregulates pro-excitotoxic TRPC3 (promoting death) but downregulates neuroprotective TRPC6	Kim et al. (2013)
TRPC7	TRPC7 deficiency blocks CA3 epileptiform bursting and synaptic LTP, regulating high-frequency excitation initiation of acute seizures	Zheng (2017)

Studies have shown that TRPC1/4/5 knockout mice exhibit a lack of epileptic-like bursts in lateral septal neurons and reduced epileptic-induced neuronal cell death; epileptic-like bursts induced by metabotropic glutamate receptor agonists in the hippocampal CA1 region were unchanged in TRPC5 knockout mice but were eliminated in TRPC1 knockout and TRPC1/4 double knockout mice. Conversely, the long-term potentiation (LTP) in TRPC5 knockout mice was significantly reduced, but LTP in TRPC1 knockout and TRPC1/4 double knockout mice remained normal. These knockout experiments suggest that TRPC5 and TRPC1/4 contribute to epilepsy and excitotoxicity through distinct cellular mechanisms (Phelan et al., 2013). In addition, mice with brain injury exhibited higher sensitivity to pentylenetetrazole-induced seizures, while subcutaneous treatment with the TRPC4 and TRPC5 inhibitors M084 significantly reduced excessive neural excitability after brain injury (Carver et al., 2021). In a rat model of epileptic seizures induced by kainate, administration of the TRPC5 inhibitor NU6027 conferred significant neuroprotection (Park et al., 2019). Pyr3, a pyrazole compound identified as a highly selective TRPC3 inhibitor that acts directly on the channel protein due to its critical trichloroacrylic amide group (Kiyonaka et al., 2009), has demonstrated efficacy in reducing both the success rate and severity of pilocarpine-induced seizures (Phelan et al., 2017), as well as mitigating seizure-induced cell death (Phelan et al., 2024). The study also found that TRPC3 was significantly elevated in febrile seizures induced in the rat hippocampus. Additionally, direct microinjection of Pyr3 into the hippocampus reduced the severity and duration of seizures and significantly prevented febrile seizure-associated brain cell death and neuroinflammation (Sun et al., 2018). Additionally, the study found that TRPC6 and TRPC3 play opposite roles in neuronal death following pilocarpine-induced seizures. Following seizures, TRPC6 expression was significantly reduced in hippocampal neurons, while TRPC3 expression increased (Kiyonaka et al., 2009; Phelan et al., 2017; Sun et al., 2018; Kim et al., 2013). Therefore, it is believed that TRPC6 activation has a neuroprotective effect, while TRPC3 activation is associated with the mediation of neuronal death (Kim et al., 2013). Furthermore, intracerebroventricular injection of TRPC6 selective activator hypericin reduces hippocampal neuronal death after seizures (Kim et al., 2013). However, studies have also shown that the expression of TRPC3 and TRPC6 is increased in resected human epileptic cortex and in the hippocampus of mouse epilepsy models (Zeng et al., 2015).

**Figure 2:**
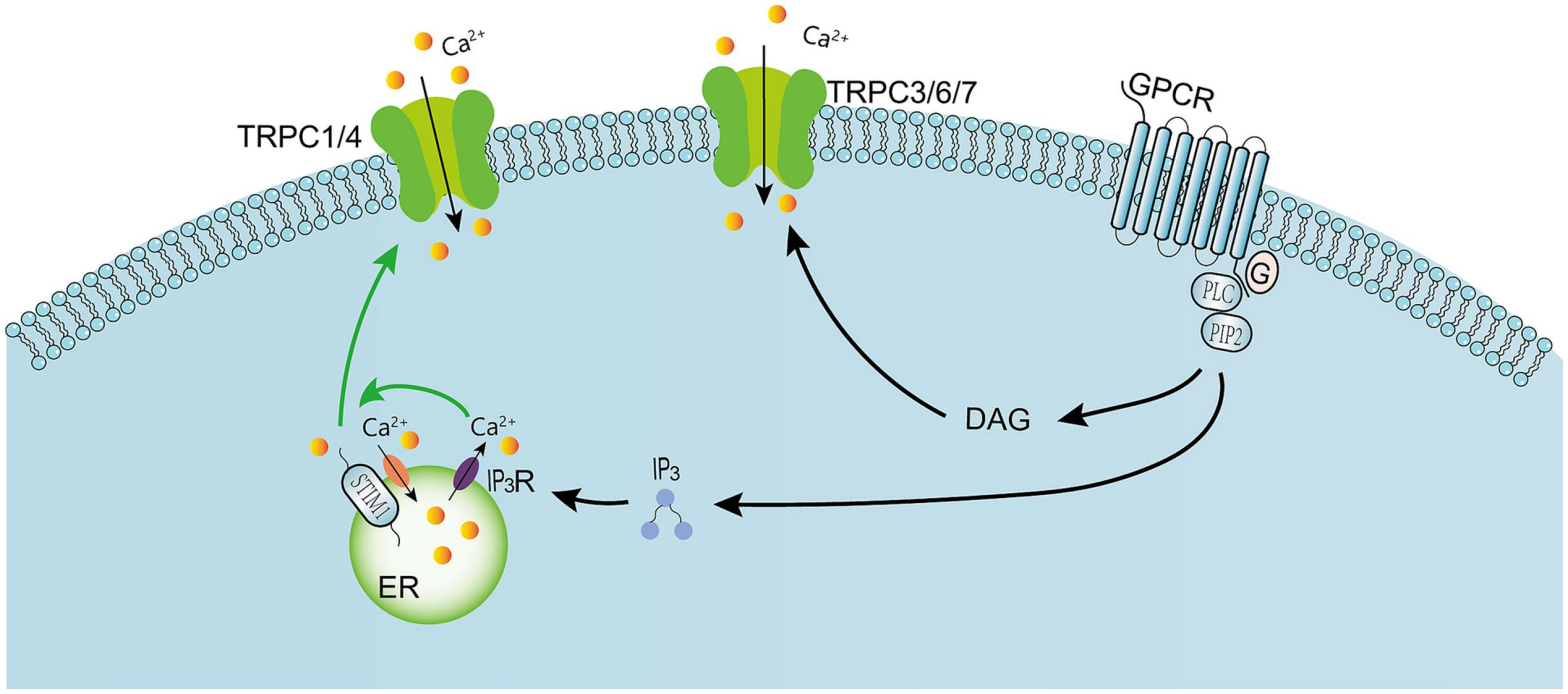
Activation of G protein-coupled receptors activates PLC, leading to the hydrolysis of PIP2 into IP3 and DAG. DAG can directly activate TRPC3, TRPC6, and TRPC7 channels (Phelan et al., 2024). IP3 binds to ligand-gated ion channels IP3R, leading to the release of Ca^2+^ from endoplasmic reticulum stores. The depletion of intracellular Ca^2+^ stores in turn allows STIMI aggregation, which subsequently activates TRPC1, 4, or ORAI Ca^2+^ channels in the plasma membrane, enabling Ca^2+^ to enter the cell. Meanwhile, calcium is pumped back into the endoplasmic reticulum via the Sarcoplasmic/Endoplasmic Reticulum Ca^2+^-ATPase (SERCA). These signaling molecules are associated with the lipid raft domain, which provides a platform for protein–protein interactions and stimulates the activation of TRPC channels. GRCP: G Protein-Coupled Receptor. IP3: Inositol 1,4,5-Trisphosphate. TRPC: Transient Receptor Potential Canonical. STIM1: Stromal Interaction Molecule 1, is an endoplasmic reticulum calcium sensor that orchestrates store-operated calcium entry (SOCE).

TRPC7 is the newest member of the TRPC subfamily and is widely expressed in peripheral tissues such as the eye, heart, kidney, lung, intestine, and pituitary gland (Zhang and Trebak, 2014). TRPC7 is also expressed in the central nervous system, but its exact function remains largely unclear. TRPC7 expression levels in the mouse hippocampus and interhemispheric commissures are lower than those of TRPC3 but higher than those of TRPC6 (Phelan et al., 2012). Knockout of the TRPC7 gene in mice significantly suppressed the induction of epileptic seizures and reduced the mortality rate of animals after pilocarpine-induced seizures. Electroencephalogram (EEG) recordings and analysis also showed that, compared with wild-type animals, pilocarpine-induced TRPC7 knockout mice exhibited relatively reduced gamma wave activity, and the decrease in seizure severity may be associated with reduced gamma wave activity (Phelan et al., 2014). Additionally, electrophysiological studies have shown that TRPC7-deficient mice exhibit reduced epileptic-like bursts in hippocampal CA3 pyramidal neurons and lack high-frequency stimulus-induced long-term potentiation at CA3 and CA1 synapses. These important findings suggest that enhanced epileptic activity in the hippocampal CA3 region, which depends on increased activity in the CA3 region, may be an early critical event in the initiation of acute epileptic seizures, with TRPC7 potentially playing a significant role in this process (Zheng, 2017). However, further research is needed to fully understand the cellular and molecular mechanisms by which TRPC7 promotes epileptic seizures (Figure 2).

## 5 The role of TRPM ion channels in epilepsy

TRPM channels constitute the most diverse and largest subfamily of the TRP superfamily, comprising eight members, TRPM1-TRPM8 (García-Rodríguez et al., 2022). Studies have found that the opening of TRPM channels is regulated by both temperature and voltage (Alvarez et al., 2019). Many TRPM channels can mediate Ca^2+^ entry into the cytoplasm (Huang et al., 2020). Similar to other TRP channels, TRPM channels have six transmembrane domains and a pore region between the fifth and sixth transmembrane domains (Huang et al., 2020). Due to the importance of maintaining Ca^2+^/Mg^2+^ homeostasis, these channels have been identified as potential targets for the treatment of neurological disorders, cardiovascular diseases, and type II diabetes (Huang et al., 2020). Research has confirmed that multiple TRPM channels are involved in the pathogenesis of epilepsy (Lee et al., 2021) (Table 3).

**Table 3:** Interaction between TRPM and epilepsy.

TRPM subfamily	Interaction	References
TRPM2	TRPM2-mediated calcium overload drives neuronal hyperexcitability and mitochondrial apoptosis, whereas EFHC1 binding potentiates oxidative vulnerability via mutation-enhanced neuronal death pathways	Zsurka and Kunz (2015), Katano et al. (2012)
TRPM7	Seizure-activated TRPM7 sustains a self-perpetuating excitotoxic-reactive oxygen species (ROS) amplification loop	Aarts and Tymianski (2005)
TRPM8	M8-B antagonism of TRPM8 reduces core temperature, protecting against febrile/PTZ seizures. TRPM8 activation may thermally sensitize pro-epileptic channels (e.g., TRPV4) to promote seizure activity	Shibasaki et al. (2020), Zandi et al. (2019)

TRPM2 is expressed in the nervous system, particularly in neurons and microglia in the hippocampus and cerebral cortex (Belrose and Jackson, 2018). Activation of TRPM2 leads to a large influx of Ca^2+^ and other cations, causing increased excitability in the epileptic brain and thereby enhancing epileptic seizures (Zsurka and Kunz, 2015). In addition, Ca^2+^ influx also leads to mitochondrial dysfunction, ultimately resulting in cell death (Zsurka and Kunz, 2015). Early research demonstrated that TRPM2 in hippocampal neurons interacts with EF-hand motif-containing protein 1 (EFHC1), disrupting neuronal apoptosis through EFHC1 mutation-mediated pathways and contributing to juvenile myoclonic epilepsy (JME) phenotypes (Katano et al., 2012). A recent study has demonstrated that multiple factors can activate TRPM2 in epilepsy, including: excessive production of reactive oxygen species, activation of the PARP (Poly ADP-ribose polymerase) signaling pathway, and increased intracellular Ca^2+^ concentration (Zheng et al., 2020). Furthermore, TRPM2 plays an important role in the pathology of epilepsy-induced cognitive impairment (Zheng et al., 2020). The study also demonstrated that congenital TRPM2 knockout in mice significantly suppressed seizures induced by pentylenetetrazole and electroconvulsive epilepsy models, reduced acute epilepsy-associated neuronal death, improved cognitive function, and alleviated brain inflammation. Importantly, the neuroprotective effects observed in these TRPM2 knockout mice may be associated with the downregulation of the PARP1/BNIP3/AIF/Endo G apoptosis pathway in cortical neurons (Zheng et al., 2020). However, another study showed that TRPM2 gene knockout in mice increased susceptibility to pentylenetetrazole-induced seizures and may enhance excitability of hippocampal CA1 neurons by inhibiting Kv7 potassium channels (Ying et al., 2022). The specific reasons for these inconsistent results are unclear, and future studies involving epilepsy gene knockout or TRPM2 drug inhibition may hold promise for resolving this issue.

TRPM3 is a non-selective cation channel activated by noxious heat (Zhao et al., 2020), The activation of this channel requires the participation of phosphatidylinositol 4,5-bisphosphate (PIP2) (Zhao and Rohacs, 2021). TRPM3 is widely expressed in various organs of the human body, particularly in neurons and oligodendrocytes in the hippocampus, locus coeruleus, cerebellum, and hypothalamus of the brain (Zhao and Rohacs, 2021). Research has found that TRPM3 can enhance glutamate transmission in Purkinje cells (Zamudio-Bulcock et al., 2011). This may explain the association between TRPM3 gene mutations and developmental and epileptic encephalopathies, a group of disorders characterized by seizures and intellectual disability (Dyment et al., 2019). Recent studies have shown that mutations in TRPM3 are frequently associated with neurodevelopmental disorders, suggesting that TRPM3 plays an important role in the immature brain (Van Hoeymissen et al., 2020). In particular, there is a strong association between mutations in the human TRPM3 gene and developmental epileptic encephalopathy (DEE) (Dyment et al., 2019; Gauthier et al., 2021; Kang et al., 2021). These mutants can be selectively inhibited by TRPM3 inhibitors such as isoflavones and primidone, the latter of which can directly inhibit TRPM3 ion channels (Krügel et al., 2017). Phenytoin is a clinically approved antiepileptic drug, and its primary mechanism of action in treating epilepsy is the inhibition of TRPM3 activity (Zhao and Rohacs, 2021). Therefore, the development of TRPM3 inhibitors is a focus for future epilepsy treatment.

**Figure 3:**
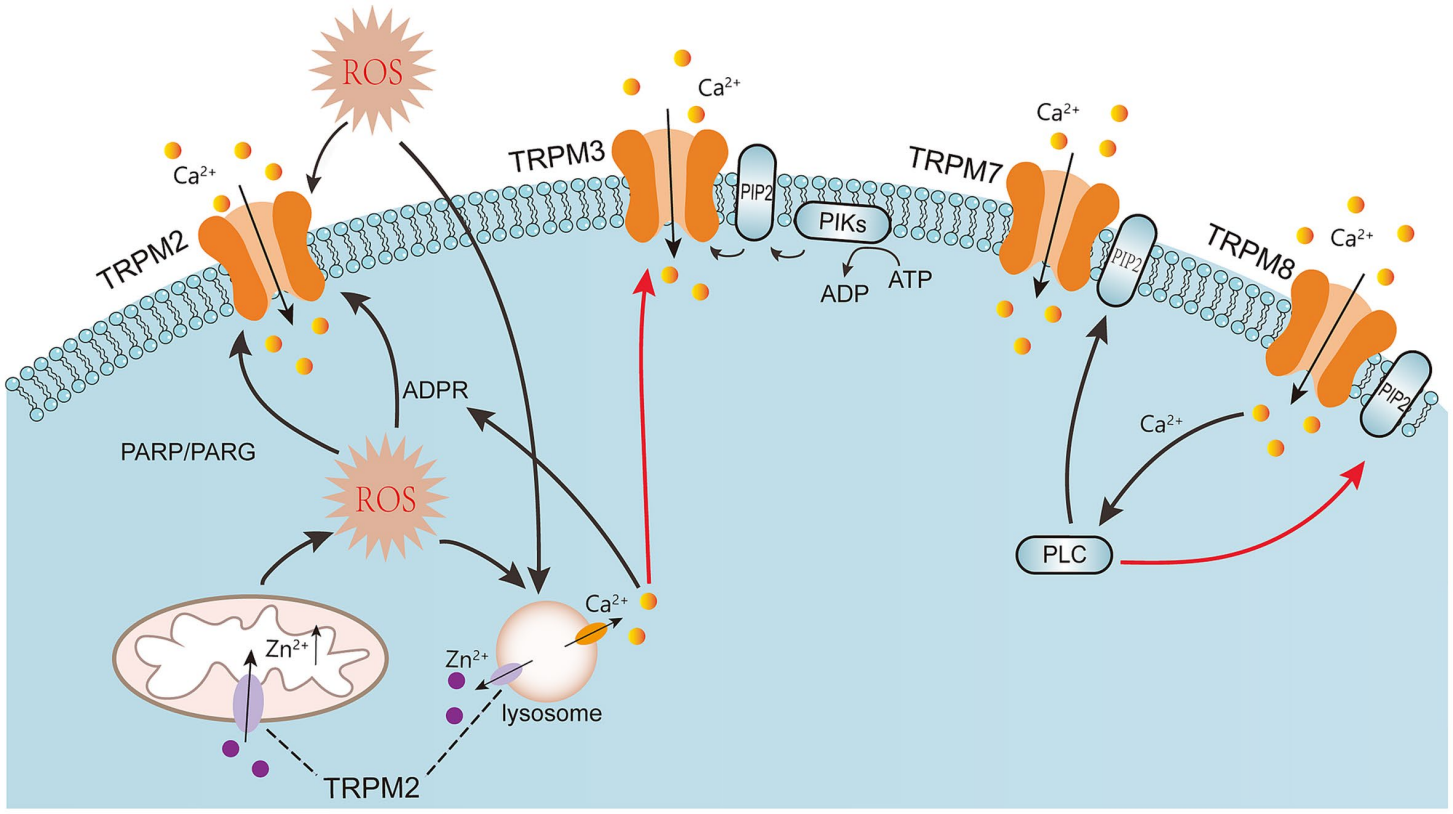
ROS generation activates TRPM2, leading to calcium ion influx. ROS-induced lysosomal dysfunction releases zinc ions, increasing intracellular zinc levels in mitochondria, which in turn causes mitochondrial dysfunction and the release of reactive oxygen species, forming a vicious cycle that results in neuronal death. Additionally, excessive reactive oxygen species can cause DNA damage, activating PARP and PARG, which subsequently activate the TRPM2 channel. TRPM3 channels can be activated by CIM0216, hypotonic solutions, and prostaglandin E2 (PS). Free calcium ions in cells can also inhibit TRPM3 channels. Naquben, prostaglandin E2, AMP, ADP, ATP, and mechanical stimuli can activate TRPM7. TRPM8 is a cold receptor that can be directly activated by low temperatures and chemical agonists (such as menthol) and regulated by key molecules (such as PIP2 and Ca^2+^). Solid black arrows represent activation, solid red arrows represent inhibition, and dashed lines represent explanatory notes. ADPR: Nicotinamide adenine dinucleotide phosphate. PARP: Poly (ADP-ribose) polymerase. PARG: Poly (ADP-ribose) glycogenase. ROS: Reactive oxygen species. TRPM: Transient receptor potential melastatin.

Central to TRPM7’s involvement in epilepsy is its unique gating, a process which is tonically inhibited by intracellular Mg·ATP. During the metabolic stress of seizures, this inhibition is relieved, permitting sustained Ca^2+^ influx that exacerbates excitotoxicity and neuronal death (Doboszewska et al., 2022; Turlova et al., 2021). TRPM7 has been shown to be activated during epilepsy, maintaining a positive feedback loop and promoting the production of reactive oxygen species (ROS); TRPM7 gene knockout can block the activation of cation currents during hypoxia and inhibit ROS-mediated cell death (Aarts and Tymianski, 2005). The study also found that TRPM7 inhibitors can reduce TRPM7 channel expression induced by epileptic seizures, decrease intracellular zinc accumulation, reduce reactive oxygen species production, and decrease neuronal death after epileptic seizures (Jeong et al., 2020). The biological function of TRPM7 remains unclear and requires further investigation TRPM8 is a non-selective channel with moderate Ca^2+^ permeability and also permeable to molecules (Mccoy et al., 2017). Unlike some of the TRP channels mentioned earlier, TRPM8 is known as the cold and menthol receptor, acting as a sensor for low temperatures and menthol (Díaz-Franulic et al., 2020; Latorre et al., 2011; Yin et al., 2018). TRPM8 exhibits its highest expression levels in the prostate, with the liver showing the next highest abundance (Fonfria et al., 2006), notable expression also occurs in the hypothalamus, hippocampus, and amygdala (Zandi et al., 2019). Research has demonstrated that TRPM8 participates in body temperature regulation, with its activation increasing core body temperature and its blockade decreasing core body temperature. This validates that the TRPM8 selective antagonist M8-B can prolong the latency period of febrile seizures in young mice and significantly inhibit pentylenetetrazole-induced seizures (Zandi et al., 2019). Therefore, the possible mechanism by which TRPM8 plays a role in epilepsy is related to temperature regulation, as channel activation increases core body temperature. Elevated temperatures may activate other TRP channels similar to TRPV4, promoting epileptic seizures (Shibasaki et al., 2020). Research has also found that menthol, a TRPM8 agonist, enhances tonic gamma-aminobutyric acid inhibition, indicating that TRPM8 plays an important role in regulating neuronal activity (Zhang et al., 2008). The study also found that menthol exerts its unique antiepileptic effect in prefrontal cortical pyramidal neurons by blocking sodium channels, a mechanism similar to that of carbamazepine, which inhibits voltage-gated sodium channels (Szulczyk and Spyrka, 2022). Additionally, TRPM8 gene knockout in mice exacerbated pentylenetetrazole-induced seizures and penicillin G potassium-induced epileptiform discharges; whereas in wild-type animals, intracortical microinjection of the TRPM8 agonist WS-3 significantly suppressed both of these conditions (Moriyama et al., 2021). These findings suggest that TRPM8 channel research may provide new strategies for controlling epileptic seizures (Figure 3).

## 6 Recent advances in TRP channel-targeted therapy

In the field of epilepsy treatment, research into targeted therapies for various subtypes of TRP channels continues to achieve new breakthroughs. Khalil et al. (Khalil et al., 2023) demonstrated that the TRPM7 inhibitors carvacrol and waixenicin A completely suppressed seizure-like activity in rodent hippocampal-entorhinal cortical slices. Llanos et al. (Llanos et al., 2022) Using virtual screening, novobiocin, montelukast, and cinnamaldehyde were identified as compounds capable of regulating TRPV1 channels and exhibiting anticonvulsant activity. Additionally, fluniprazine reduced the total duration of glutamatergic responses by blocking TRPM4 channels, epileptic activity can be eliminated (Sinyak et al., 2024). The novel pyrazole compound JW-65 significantly reduced the frequency and severity of epileptic seizures in mice by inhibiting TRPC3 channels (Nagib et al., 2022). The antiepileptic effects of endogenous cannabinoid metabolites anandamide (AEA) and 2-arachidonoylglycerol (2-AG) are also associated with TRP channels (Jacobs and Sehgal, 2020). These research findings reveal the enormous potential of TRP channels in epilepsy treatment, providing new targets and directions for the development of more effective epilepsy drugs in the future.

## 7 Conclusion

Epilepsy is a condition characterized by recurrent synchronous neuronal discharges that disrupt normal neuronal function, believed to result from abnormal brain electrical activity. Increasing research has identified voltage-gated Ca^2+^ channels as playing roles in various processes including neuronal proliferation and differentiation, membrane excitability, signal transduction, gene expression, neurotransmitter release, axon growth, and synaptogenesis. Ca^2+^ signaling is increasingly recognized as a key factor in the onset of epilepsy. Intracellular Ca^2+^ homeostasis involves multiple Ca^2+^-permeable cation channels, with TRP channels potentially being the most important. Furthermore, reactive oxygen species (ROS) originating from NADPH oxidases and other non-neuronal sources also significantly influence TRP channel function (Miller, 2006), potentially contributing to Ca^2+^ dyshomeostasis and epileptogenesis. Research on TRP channels may identify multiple potential drug targets and elucidate their possible biological mechanisms, thereby clarifying the role of TRP channels in the diagnosis and treatment of epilepsy.
